# Efficacy and Safety of Artesunate-Amodiaquine versus Artemether-Lumefantrine in the Treatment of Uncomplicated *Plasmodium falciparum* Malaria in Sentinel Sites across Côte d'Ivoire

**DOI:** 10.1155/2015/878132

**Published:** 2015-08-12

**Authors:** William Yavo, Abibatou Konaté, Fulgence Kondo Kassi, Vincent Djohan, Etienne Kpongbo Angora, Pulcherie Christiane Kiki-Barro, Henriette Vanga-Bosson, Eby Ignace Hervé Menan

**Affiliations:** ^1^Faculty of Pharmacy, Department of Parasitology and Mycology, Félix Houphouët-Boigny University, BPV 34, Abidjan, Côte d'Ivoire; ^2^Malaria Research and Control Centre, National Institute of Public Health, BPV 47, Abidjan, Côte d'Ivoire; ^3^Parasitology and Mycology Laboratory of Diagnosis and Research Centre on AIDS and Opportunistic Diseases, 01 BPV 13, Abidjan, Côte d'Ivoire

## Abstract

Two years after the introduction of free Artesunate-Amodiaquine (ASAQ) and Artemether-Lumefantrine (AL) for the treatment of uncomplicated malaria in public health facilities in Côte d'Ivoire, we carried out this study to compare their efficacy and tolerability in three surveillance sites. It was a multicentre open randomised clinical trial of 3-day ASAQ treatment against AL for the treatment of 2 parallel groups of patients aged 2 years and above. The endpoints were (1) Adequate Clinical and Parasitological Response (ACPR) at day 28 and (2) the clinical and biological tolerability. Of the 300 patients who were enrolled 289, with 143 (49.5%) and 146 (50.5%) in the ASAQ and AL groups, respectively, correctly followed the WHO 2003 protocol we used. The PCR-corrected ACPR was 99.3% for each group. More than 94% of patients no longer showed signs of fever, 48 hours after treatment. Approximately 78% of the people in the ASAQ group had a parasite clearance time of 48 hours or less compared to 81% in the AL group (*p* = 0.496). Both drugs were found to be well tolerated by the patients. This study demonstrates the effectiveness and tolerability of ASAQ and AL supporting their continuous use for the treatment of uncomplicated *P. falciparum* malaria infection in Côte d'Ivoire.

## 1. Background

Malaria remains a serious health concern in sub-Saharan Africa [1]. In Côte d'Ivoire, malaria accounts for 43% of outpatient visits with one-third of reported death in health facilities [2]. Due to the development and increasing resistance of* P. falciparum* to various antimalarial drugs available, the WHO introduced and recommended the use of artemisinin-based combination therapy (ACT) for the treatment of malaria [3, 4].

This recommendation was adopted by Côte d'Ivoire in 2007 [5]. Since 2010, Artesunate-Amodiaquine (ASAQ) and Artemether-Lumefantrine (AL) are given freely in public health facilities to malaria patients to reduce malaria morbidity and mortality. Although ACTs should only be prescribed following a positive malaria test and not clinical observation, this prerequisite is not always accomplished (Yavo et al., unpublished data). Thus, there is a risk of selection of* P. falciparum* resistant strains as a result of drug pressure. Furthermore, self-medication, poor adherence to treatment, counterfeit drugs, and human and* Plasmodium* genetic makeup may influence the efficacy and safety profiles of ACTs in Côte d'Ivoire.

Indeed, resistance of* P. falciparum* to artemisinin derivatives has been recently documented in the Cambodia-Thailand border region [6, 7]. This region is an epicentre of malaria resistance worldwide. Indeed, malaria parasites that are highly resistant to chloroquine and pyrimethamine spread from Asian origins to Africa, a great cost to human health and life. If artemisinin-resistant falciparum malaria follows the same pattern, renewed efforts to eliminate and eradicate malaria will be gravely threatened [8, 9]. It is therefore necessary to implement an improved program for monitoring drug-resistant malaria in order to plan and adopt appropriate strategies to control this disease.

The aim of this study was to compare the efficacy and tolerability of Artesunate-Amodiaquine and Artemether-Lumefantrine for the treatment of uncomplicated falciparum malaria two years after their large-scale use in Côte d'Ivoire as first-line and second-line treatment.

## 2. Methods

### 2.1. Study Site

The study was carried out between June and September 2012 in three surveillance sites for antimalarial drug efficacy in Côte d'Ivoire: Abengourou (forest zone), San Pedro (coastal and forest zone), and Yamoussoukro (forest transition zone). In each site, two popular and well known health centres were chosen for the survey. Throughout the country, there are four distinct seasons divided into two raining seasons (December–July and October-November) with high malaria transmission and two dry seasons (December–March and August-September).

### 2.2. Study Design

A controlled randomized multicentre and open therapeutic trial with a 28-day follow-up period comparing the efficacy, safety, and tolerability of two Fixed-Dose Combinations (FDC) which are ASAQ and AL in patients from 2 years old and above was used. Throughout the survey, the standard WHO 2003 efficacy assessment protocol was followed [10].

### 2.3. Sample Size Determination

Based on previous studies [11–13], the proportion of probable clinical failures with ACTs investigated would not be greater than 10% with a confidence interval of 95% and a precision size of 10%. Based on an assumption of 90% efficacy for both ACTs, 6% of noninferiority margin, 80% margin power, and a one-sided 5% significance level, we calculated the number of patients required per site and per study arm. Taking into account a 10% lost to follow-up rate, the total number of patients was rounded to 60 per study arm. However, due to budget constraints, the number was maintained at 100 patients per site (50 per each arm).

### 2.4. Study Population

The study population consisted of outpatients who came to the health facilities with uncomplicated malaria-like symptoms. Patients were referred to the study team for recruitment. Inclusion criteria for the study were as follows: (1) being at least two years old; (2) fever with axillary temperature ≥37.5°C; (3)* P. falciparum* monoinfection with parasitaemia from 2,000 to 200,000/*μ*L of blood. Patients with signs or evidence of severe malaria/malnutrition, repeated vomiting, intercurrent infectious disease, history of previous serious side effects to the drugs used during the trial, and past cardiac, hepatic, or renal history or those who were pregnant (positive test) or breast-feeding were excluded. Criteria to stop the treatment and/or withdrawal of a patient from the study included the following: (1) occurrence of serious adverse effects; (2) unsatisfactory therapeutic response; (3) violation of the protocol; (4) withdrawal of consent; and (5) being lost to follow-up. Before inclusion, written informed consent was obtained from the patient or the patient's legal guardian. Approval was obtained from the national ethics committee before study onset.

### 2.5. Study Procedures

For each patient involved in the study, the protocol was read and explained to him/her or to the legal guardian (as regards children). On acceptance, patient or legal guardian had to sign the informed consent forms to take part in the study. For all the recruited patients, baseline examinations and laboratory investigations were conducted immediately and free of charge. Those among the patients who met inclusion criteria at baseline were randomly assigned to one of the two treatment groups following a randomization list. In each study site, computer generated randomization codes were prepared by an independent individual. These codes were enclosed in sequentially numbered opaque sealed envelopes, each of which contained the treatment allocation. The envelopes were assigned in sequential order to participants after inclusion.

### 2.6. Therapeutic Groups

#### 2.6.1. Study Treatment

Each patient was allocated to one of the two treatment groups. Artesunate- (AS) Amodiaquine (AQ) (ASAQ) (Winthrop, Sanofi-Aventis, France) was administered as a single daily dose for three days. Each tablet of ASAQ contained either 50 mg of AS and 135 mg of AQ or 100 mg of AS and 270 mg of AQ. ASAQ treatments varied according to body weight: 9–17 kg, one tablet (50 mg/135 mg) per dose; 18–36 kg, one tablet (100 mg/270 mg) per dose; and over 36 kg, two tablets (100 mg/270 mg) per dose. Artemether-Lumefantrine (AL) tablets (Ipca, Laboratories, India) were administered at 0 and 8 hours on day 1 and then twice daily for two subsequent days according to body weight: 5–14 kg, one tablet per dose; 15–24 kg, two tablets per dose; 25–34 kg, three tablets per dose; 35 kg and over, four tablets per dose. All treatments were given under direct supervision of a member of the study team. If the patient vomited within 30 minutes after taking the drug, the whole dose was readministered. However, if the vomiting persisted, the patient was removed from the study and referred to the health centre for an in-depth investigation and treatment according to the current national policy. The dose could not be administered again if vomiting occurred more than 60 minutes after administration.

#### 2.6.2. Concomitant Treatment

Concomitant treatment refers to the treatment of diseases other than malaria. Antipyretic and antiallergic were provided when needed during the follow-up.

Antibiotics such as sulphonamide, tetracycline, quinolone, and macrolide were contraindicated during the study because of their possible activity against* Plasmodium* which could interact or lead to false evaluation of the drug efficacy evaluation.

### 2.7. Follow-Up

After inclusion, patients were scheduled for follow-up examinations on days 1, 2, 3, 4, 7, 14, 21, and 28 using WHO in vivo tests with a follow-up period of 28 days [10, 14]. These examinations should be done at any other time, if the participant felt unwell during the study period. On each visit, physical and clinical examinations and biological evaluations were performed. Patients or guardians were also asked about drug adverse effects. Patients who failed the treatment were given quinine or artemether infusion according to the national treatment guidelines. Blood samples were also collected for* P. falciparum* molecular biology analysis at baseline and then after day 7 in case of parasitaemia. The response to treatment was measured and defined according to WHO guidelines [10].

### 2.8. Investigations

#### 2.8.1. Laboratory Methods

At each visit, thick and thin blood films were performed. The density of* P. falciparum* in the peripheral blood was determined by counting the number of asexual parasites in 200 white blood cells (WBC). All of the thick and thin blood films were reread to double check. A slide was considered negative after reading 200 microscopic fields. The presence of gametocytes was also noted. In case of discrepancy, a third reading was made by a third microscopist. An external quality control was carried out on 10% of the slides. Venous blood was collected on days 1 and 4 for conducting haematological (full blood count) and biochemical (creatinine, AST, ALT, and total bilirubin) investigations.

#### 2.8.2. Parasite Genotyping

In order to distinguish recrudescence from new infection, filter-paper blood spots (Whatman International Ltd., Maidstone, UK) were collected from finger pricks on day 1 and on the day of recurrent parasitaemia (after day 7) and used for molecular genotyping. Parasite DNA was extracted from filter-paper blood spots using the Chelex methods [15] and analysed for length polymorphisms in the gene encoding merozoite surface protein-1 (*msp1*) and merozoite surface protein-2 (*msp2*) using nested PCR as described by Soulama et al. [16].

### 2.9. End Points

#### 2.9.1. Efficacy Evaluation

Consider the following:

- Primary efficacy parameter was the cure rate at day 28. It is the proportion of patients for whom removal parasitaemia is obtained within 7 days of the study without recrudescence within 28 days after the start of study treatment. The recurrence is defined as a new clinical manifestation of the infection after initial removal of parasites in the peripheral blood. However, in case of reinfection (verified by PCR), parasitological recurrence is not considered as a treatment failure of malaria drug received.
- Secondary efficacy endpoints were as follows: (a) Cure rate at 14 days: proportion of patients for whom removal parasitaemia is obtained within 7 days of the study without recrudescence within 14 days after the start of study treatment. (b) Parasite clearance time: time elapsed between the first administration and the first total and continued disappearance of parasite asexual forms and persisting for at least another 24 hours. (c) Thermal clearance time: time elapsed between the first dose and the first lowering of the temperature below 37.5°C for at least another 24 hours. (d) Gametocyte carriage evolution. (e) Improvement in haemoglobin rate compared to the start of the study.

#### 2.9.2. Tolerability Evaluation

It consisted of monitoring and registration of any adverse event (date of onset, severity, and duration), biological monitoring (haematological, biochemical), and the assessment of the clinical status of subject (vital signs, physical examinations) during follow-up. Any clinical or biological sign not present in inclusion and which appeared during follow-up or any sign present at day 1 and worsening thereafter was considered as adverse event.

### 2.10. Statistical Analysis

All data were recorded and checked using Epi data version 3.1 and analysed with SPSS for windows (version 16.0). The characteristics of patients in the two groups at inclusion were compared using Pearson's Chi-square test and independent samples *t*-test. The cases of protocol violation and withdrawn consent were censored at the time they left the study. The distributions of fever and parasite clearance were compared using Pearson's Chi-square test. Differences of haemoglobin and biochemical parameters values within individuals between day 1 and day 4 were computed. Changes in haemoglobin concentrations and in biochemical parameters were compared using the paired *t*-test. The level of significance for statistical tests was set at 0.05. Data were done in Per Protocol (PP) analysis.

## 3. Results

### 3.1. Global Distribution of Patients in the Study

**Figure 1:**
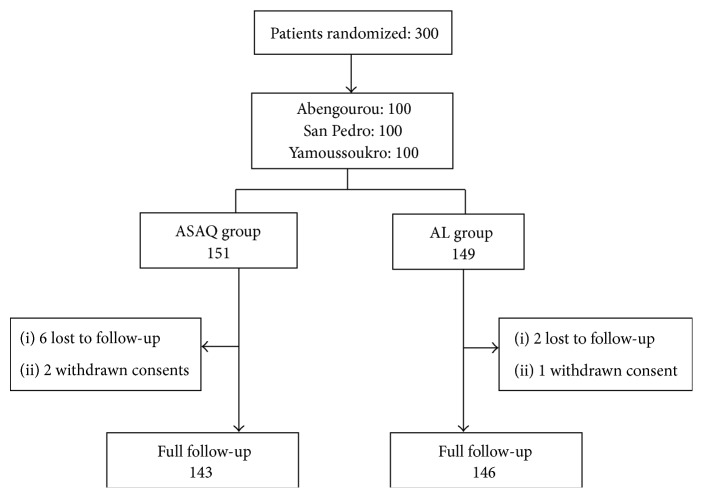
Trial profile.

A total of 300 patients were included in the study. 151 patients were randomized to ASAQ and 149 to AL. In the ASAQ group, six patients were lost to follow-up and two patients have withdrawn their consent. In the AL group, there were two patients lost to follow-up and one case of consent withdrawal. Finally, 143 (49.5%) patients and 146 (50.5%) patients were successfully followed up, respectively, in ASAQ and AL groups (Figure 1).

### 3.2. Baseline Characteristics of Patients

Baseline characteristics of patients receiving either ASAQ or AL are summarized in Table 1. The distribution by gender and age as well as the average temperatures in the two treatment groups did not show any statistical significant differences. Other biological parameters (haematological and biochemical) also followed the same trend.

### 3.3. Primary Outcome: Day 28 Cure Rates for Both Treatments

The results of the treatment efficacy are presented by treatment group, day of follow-up, unadjusted and adjusted by genotyping, and age of study patients (Table 2). Cure rates decreased during follow-up in the two treatment groups. Nevertheless, at day 28 before PCR correction, ASAQ was highly effective in the treatment of* P. falciparum* infections and preventing parasite recurrences compared to AL. Only nine treatment failures were observed: 1 (0.7%) in the ASAQ group and 8 (5.5%) in the AL group. Most of the therapeutic failures were classified as LCF. And most of LCF cases were found among children between five and fifteen (6.8%). Any case of Late Parasitological Failure (LPF) was observed. After PCR correction, both treatments had the same Adequate Clinical and Parasitological Response (ACPR) (99.3%).

### 3.4. Secondary Efficacy Outcomes

#### 3.4.1. Day 14 Cure Rate for Both Treatment

At day 14, cure rate was 100% with ASAQ and 99.3% with AL. Indeed, one case of Early Treatment Failure (ETF) was observed in AL group in an under-five child.

#### 3.4.2. Parasite and Fever Clearance

**Figure 2:**
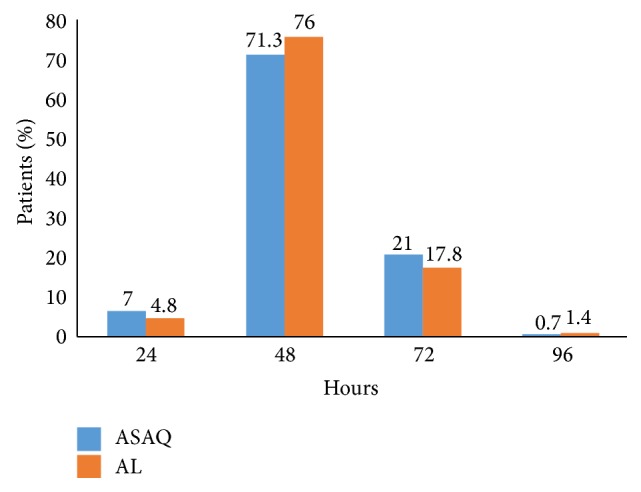
Parasite clearance in the two groups. ^*∗*^ Pearson's Chi-squared test; *p* value = 0.496.

Parasite clearance on days 1 and 2 was similar in both groups. Most of the patients of the two treatment groups had a parasite clearance ≤ 48 hours. Difference in parasite clearance between the two therapeutic groups was not statistically significant (*p* = 0.496) (Figure 2).

**Figure 3:**
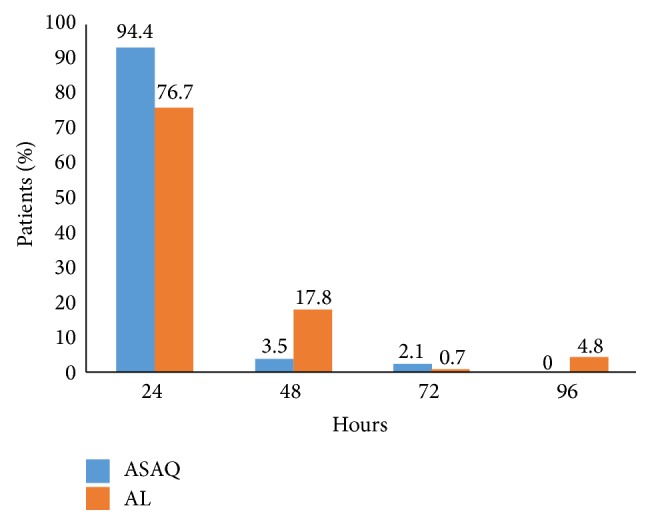
Fever clearance in the two groups. ^*∗*^ Pearson's Chi-squared test; *p* value = 0.00086.

In both treatment groups, the majority of subjects had fever clearance ≤ 24 hours. Beyond 72 hours, all the patients treated by ASAQ were nonfebrile. Seven patients in the AL group were febrile after 96 hours (Figure 3). The difference observed in the distribution of thermal clearance between the two groups was statistically significant (*p* = 0.00086).

#### 3.4.3. Gametocyte Carriage

**Figure 4:**
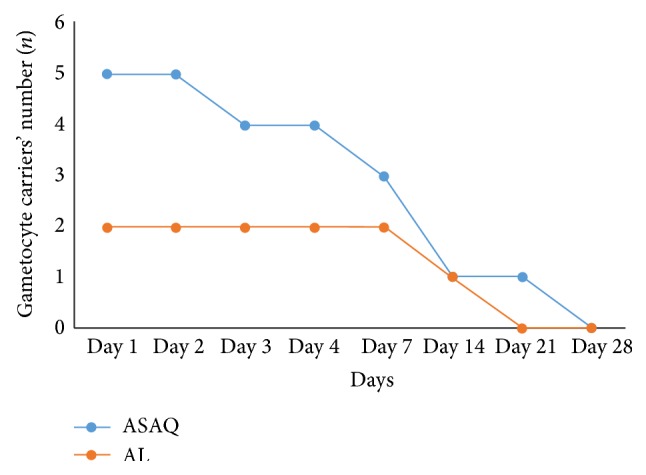
Changing patterns for gametocyte carriers' number.

At inclusion, there were only two gametocyte carriers in the ASAQ group and five in the AL group. In both therapeutic groups, the number of gametocyte carriers decreased throughout the follow-up and was zero at day 28 (Figure 4).

### 3.5. Tolerability Evaluation

#### 3.5.1. Clinical Level

Among the 289 patients followed up, in 175 (121 ASAQ and 54 AL) some side effects were observed (60.5%). Generally, adverse events were regular in the ASAQ group and included pruritus, asthenia, and drowsiness and vomiting. The AL seemed clinically better tolerated than ASAQ. However, side effects were not severe in both treatments as to interrupting the treatment (Table 3).

#### 3.5.2. Biological Level

Decrease in haemoglobin values was observed from the inclusion to day 4. This decrease was more significant in AL group (−1.02 g/dL) than ASAQ group (−0.86 g/dL). The decrease was significant within both groups (*p* < 0.001). Between day 1 and day 4 in both treatment groups we observed that the mean of ALT decreased but was not significantly different, the level of creatinine varied but not significantly, and finally the amount of bilirubin decreased significantly in both treatment arms (*p* < 0.001) (Table 4).

## 4. Discussion

This randomized trial enabled an evaluation of the efficacy and tolerability of ASAQ versus AL in three sentinel sites in Côte d'Ivoire as part of the uncomplicated* P. falciparum* malaria treatment among patients more than two years old.

ASAQ appeared to be a better treatment option on the basis of non-PCR-corrected responses, based on the lower percentage of recurrent parasitaemia observed. However, the PCR-corrected cure rates which indicated the true efficacy were the same for both treatments. The results showed a high cure rate for both regimens after a standard 28-day follow-up with an ACPR rate adjusted by 99.3%. This is consistent with efficacy results reported from several sub-Saharan African countries [11–13, 17–23].

The fact that there was more reinfection than recrudescence shows that malaria transmission is high in all three sentinel sites [13]. Indeed, in Côte d'Ivoire, malaria is perennial with a peak during rainy seasons. According to the situational analysis made by the National Malaria Control Program (NMCP) in 2010, malaria accounts for 43% of all causes of outpatient visits [2]. Some studies which have compared ASAQ with AL over a follow-up duration of 28 days or longer have demonstrated a lower reinfection rate for AL and found that AL was superior to ASAQ in preventing new infections [18, 20, 22]. However, our results are in contrast to these studies. The LCF rate until day 28 uncorrected was frequently observed in AL-treated patients than in ASAQ-treated individuals. In a study carried among Ghanaian children, the authors obtained the same assertion with the same ACPR rate. This could reflect a difference in the prevention of recurrent clinical malaria episodes between the two ACTs [24]. Moreover, studies that have compared AL with ACT regimens consisting of longer-acting partner drugs have demonstrated a shorter time to reinfection for AL [25–27]. The follow-up period remains a limitation of our study, with the failure rate at 42-day or 63-day follow-up period reported as real.

Fever clearance was fast in both treatment groups confirming the previous data [28]. However, beyond 72 hours, all the patients treated by ASAQ were nonfebrile unlike those treated with AL. Prompt parasite clearance times by both AL and ASAQ groups are in agreement with findings from Africa and elsewhere [29].

The decrease of gametocyte rate during the treatment with ACTs has been demonstrated. This action decreases transmission and therefore leads to a significant reduction in the spread of resistance [30, 31]. In this study, the number of gametocyte carriers decreased throughout follow-up.

The clinical tolerance was good with minor adverse events in both treatment groups confirming previous studies [11]. Regarding biological aspects, the haemoglobin rate decrease was more important in AL group and this suggests that convalescence is obtained faster in the ASAQ treatment group. Moreover, a significant increase in the number of patients presenting anaemia was observed in both groups confirming previous studies [11, 32]. The bilirubin decrease was significant in both groups suggesting that liver function has been improved significantly in both treatment groups [11].

## 5. Conclusion

During our study, we found that ASAQ was as effective and well-tolerated as was AL in the treatment of uncomplicated* falciparum* malaria. Therefore, this work supports the continued use of these ACTs in the management of malaria with added advantage provided in public health facilities in slowing the spread of malaria drug resistance and of global reduction or elimination of malaria in Côte d'Ivoire.

## Figures and Tables

**Table 1:** Baseline characteristics of recruited patients (Day 1).

	ASAQ	AL	*p* ^*∗*^	Global
	143	146		
Number of patients				289

Sex				
M, *n* (*%*)	63 (*44.1*)	71 (*48.6*)	*0.508 *	134 (*46.4*)
F, *n* (*%*)	80 (*55.9*)	75 (*51.4*)		155 (*53.6*)

Mean age (SD) years	8.87 (*9.09*)	8.06 (*6.72*)	*0.389 *	8.46 (7.98)
Min-max	2–63	2–52		2–63
[2–5[, *n* (*%*)	69 (*48.3*)	62 (*42.5*)		131 (*45.3*)
[5–15[, *n* (*%*)	57 (*39.9*)	73 (*50.0*)		130 (*45.0*)
[15–63], *n* (*%*)	17 (*11.9*)	11 (*7.5*)		28 (*9.7*)

Mean temperature (SD) °C	38.8 (0.84)	38.9 (0.94)	*0.341 *	38.84 (0.89)
Min-max	37.5–44.6	37.5–41.2		37.5–41.2
[37,5–38,5[, *n* (*%*)	62 (*43.4*)	63 (*43.2*)		125 (*43.3*)
[38,5–41,2], *n* (*%*)	81 (*56.6*)	83 (*56.8*)		164 (*56.7*)

Mean parasitaemia (SD) tpz/*μ*L	51200 (58259)	51300 (56603)	*0.963 *	51200 (57328)
(écart-type) tpz/*μ*L Min-max	2108–200000	2000–200000		2000–200000

Gametocyte carrier rate, *n* (*%*)	5 (*3.50*)	2 (*1.37*)	*0.428 *	7 (*2.42*)

Mean AST (SD) UI/L	34.66 (19.40)	36.50 (20.43)	*0.433 *	35.59 (19.92)
Min-max	6–109	4–153		4–153

Mean ALT (SD) UI/L	23.01 (14.26)	23.07 (13.96)	*0.971 *	23.04 (14.09)
Min-max	5.29–88	5–87.6		5–88

Mean creatinine (SD) mg/L	8.45 (2.97)	8.00 (2.18)	*0.143 *	8.22 (2.61)
Min-max	4.00–21.87	3.95–13.72		3.95–21.87

Mean bilirubin (SD) mg/L	9.23 (7.65)	8.66 (8.71)	*0.555 *	8.94 (8.20)
Min-max	1–38.64	1.07–53.57		1–53.57

Mean haemoglobin (SD) g/dL	10.00 (1.75)	10.02 (1.89)	*0.926 *	10.01 (1.82)
Min-max	6–14.3	6–19		6–19

^*∗*^Independent samples *t*-test.

**Table 2:** PCR-uncorrected and PCR-adjusted days 14 and 28 treatment outcomes according to study participant ages.

Age (year)	ASAQ *N* (%)				AL *N* (%)			
	[2–5[	[5–15[	[15–63]	Total	[2–5[	[5–15[	[15–63]	Total
PCR-uncorrected day 14 cure rates								
ACPR	69/69 (100)	57/57 (100)	17/17 (100)	**143/143 (100)**	61/62 (98.4)	73/73 (100)	11/11 (100)	**145/146 (99.3)**
ETF	0/69 (0)	0/57 (0)	0/17 (0)	**0/143 (0)**	1/62 (1.6)	0/73 (0)	0/11 (0)	**1/146 (0.7)**
LCF	0/69 (0)	0/57 (0)	0/17 (0)	**0/143 (0)**	0/62 (0)	0/73 (0)	0/11 (0)	**0/146 (0)**

PCR-uncorrected day 28 cure rates								
ACPR	68/69 (98.5)	57/57 (100)	17/17 (100)	**142/143 (99.3)**	59/62 (95.2)	68/73 (93.2)	11/11 (100)	**138/146 (94.5)**
ETF	0/69 (0)	0/57 (0)	0/17 (0)	**0/143 (0)**	1/62 (1.6)	0/73 (0)	0/11 (0)	**1/146 (0.7)**
LCF	1/69 (1.4)	0/57 (0)	0/17 (0)	**1/143 (0.7)**	2/62 (3.2)	5/73 (6.8)	0/11 (0)	**7/146 (4.8)**

PCR-corrected day 28 cure rates								
ACPR	68/69 (98.6)	57/57 (100)	17/17 (100)	**142/143 (99.3)**	61/62 (98.4)	73/73 (100)	11/11 (100)	**145/146 (99.3)**
ETF	0/69 (0)	0/57 (0)	0/17 (0)	**0/143 (0)**	1/62 (1.6)	0/73 (0)	0/11 (0)	**1/146 (0.7)**
LCF	1/69 (1.4)	0/57 (0)	0/17 (0)	**1/143 (0.7)**	0/62 (0)	0/73 (0)	0/11 (0)	**0/146 (0)**

**Table 3:** Adverse events frequency.

	ASAQ		AL		Total *n* (%)	Fisher's exact test
	*n*	%	*n*	%		*p* value
Pruritus	42	29.4	5	3.4	47 (16.3)	<0.001
Asthenia	37	25.9	12	8.2	49 (16.9)	0.255
Vomiting	15	7.7	11	10.3	26 (9.0)	0.171
Drowsiness	14	9.8	1	0.7	15 (5.2)	0.034
Abdominal pain	5	3.5	7	4.8	12 (4.1)	0.375
Cough	3	2.1	6	4.1	9 (3.1)	0.017
Dizziness	2	1.4	1	0.7	3 (1.0)	0.925
Nausea	1	0.7	5	3.4	6 (2.0)	0.005
Diarrhoea	1	0.7	5	3.4	6 (2.0)	0.005
Hypoglycaemia	1	0.7	0	0	1 (0.3)	0.503
Insomnia	0	0	1	0.7	1 (0.3)	0.133
Total	**121**	**84.6**	**54**	**37**	**175 (60.5)**	

**Table 4:** Changing patterns for biological parameters in the two groups.

	ASAQ				AL			
	D1	D4	D1–D4	*p* ^*∗*^	D1	D4	D1–D4	*p* ^*∗*^
Haemoglobin (g/dL) (SD)	10 (1.75)	9.14 (1.78)	0.86 (−0.03)	0.000045	10 (1.89)	8.98 (1.85)	1.02 (0.04)	0.0001
AST (IU/L) (SD)	34.66 (19.4)	29.79 (18.58)	4.87 (0.82)	0.031	36.50 (20.43)	35.44 (67.57)	1.06 (−47.14)	0.533
ALT (IU/L) (SD)	33.01 (14.26)	22.46 (14.57)	10.55 (−0.31)	0.747	23.07 (13.96)	21.61 (12.12)	1.46 (1.84)	0.344
Bilirubin (mg/L) (SD)	9.23 (7.65)	5.89 (5.07)	3.34 (2.58)	0.0002	8.66 (8.71)	5.49 (5.22)	3.17 (3.49)	0.0002
Creatinine (mg/L) (SD)	8.44 (2.96)	8.51 (2.92)	−0.07 (0.04)	0.840	8.0 (2.18)	7.49 (2.26)	0.51 (−0.08)	0.056

^*∗*^Paired *t*-test.

## Abbreviations

ASAQ: Artesunate-Amodiaquine
AL: Artemether-Lumefantrine.
